# SMPD3 deficiency perturbs neuronal proteostasis and causes progressive cognitive impairment

**DOI:** 10.1038/s41419-018-0560-7

**Published:** 2018-05-03

**Authors:** Wilhelm Stoffel, Britta Jenke, Inga Schmidt-Soltau, Erika Binczek, Susanne Brodesser, Ina Hammels

**Affiliations:** 1 Laboratory of Molecular Neuroscience, Institute of Biochemistry, University of Cologne, 50931 Cologne, Germany; 2 CMMC (Centre for Molecular Medicine), University of Cologne, 50931 Cologne, Germany; 3 CECAD (Cluster of Excellence: Cellular Stress Responses in Aging-Associated Diseases), University of Cologne, 50931 Cologne, Germany

## Abstract

Neutral sphingomyelinase *smpd3* is most abundantly expressed in neurons of brain. The function of SMPD3 has remained elusive. Here, we report a pathogenetic nexus between absence of SMPD3 in the Golgi compartment (GC) of neurons of the *smpd3-/-* mouse brain, inhibition of Golgi vesicular protein transport and progressive cognitive impairment. Absence of SMPD3 activity in the Golgi sphingomyelin cycle impedes remodeling of the lipid bilayer, essential for budding and multivesicular body formation. Importantly, we show that inhibition of the Golgi vesicular protein transport causes accumulation of neurotoxic proteins APP, Aβ and phosphorylated Tau, dysproteostasis, unfolded protein response, and apoptosis, which ultimately manifests in progressive cognitive decline, similar to the pathognomonic signatures of familial and sporadic forms of Alzheimer´s disease. This discovery might contribute to the search for other primary pathogenic mechanisms, which link perturbed lipid bilayer structures and protein processing and transport in the neuronal Golgi compartment and neurodegeneration and cognitive deficits.

## Introduction

Sphingomyelin (SM) is a major component of the lipid bilayer of subcellular membranes particularly of mammalian central nervous system (CNS). Acid (SMPD1) and neutral sphingomyelinases (sphingomyelin phosphor-diesterases, SMases) (SMPD2 and SMPD3), hydrolyze SM and release ceramide and phosphoryl-choline. SMases differ in their enzymatic properties, regulation, tissue distribution, and subcellular localization. SM is degraded constitutively by acid SMase (SMPD1) in lysosomes^1–4^. Genetic defects in *smpd1* lead to the fatal human neurovisceral form of Niemann–Pick disease, type A, characterized by lysosomal SM storage in cells of the reticuloendothelial system and neurons. The SMPD1-deficient mouse mutant is a mimicry of NPD type A^5,6^. SMPD1 activity exceeds non-lysosomal neutral SMPD2 activity (nSMase1) in the endoplasmic reticulum (ER) and of SMPD3 in the GC in all tissues except brain^7^. Ligand cell surface receptor mediated activation of nSMases is believed to trigger the breakdown of SM to ceramide, acting as lipid-signaling molecule in multiple cellular signaling pathways ranging from cell growth, differentiation and apoptosis, anti-apoptosis, tumor-suppressive, and anti-proliferative cellular processes to cell senescence^8–11^. However, new aspects on the role of nSMases arose from the discovery and characterization of two Mg^2+^-dependent nSMases, SMPD2, and SMPD3^12–16^. SMPD3 is most abundantly expressed in brain. The *smpd3-null* allellic mouse model has been instrumental in defining the enigmatic systemic and cell-specific role in extra-neuronal tissues and in brain. Systemic deficiency of SMPD3 results in a complex phenotype characterized by impaired secretion of proteohormones from hypothalamic neurosecretory neurons during the postnatal growth phase thereby perturbing the hypothalamus–pituitary growth axis, and resulting in combined pituitary hormone deficiency^17^.

Studies in primary chondrocyte culture provided initial mechanistic insight into SMPD3 function^18^. Chondrocytes of the epiphyseal growth plate highly express SMPD3 during the postnatal growth phase^18–20^. Primary chondrocytes of juvenile *smpd3-/-* mice revealed retarded transport and secretion of extracellular matrix proteins in the Golgi secretory pathway, impeded proteostasis, and induced ER stress, manifested in growth retardation, and chondrodysplasia. The available data of these studies suggested a novel mechanism underlying a basic function of SMPD3 in the canonical Golgi secretory pathway. This notion and the abundant expression of *smpd3* in brain motivated further studies of SMPD3 function in the CNS.

Progressive depositions of neuronal proteins characterize several age-related neurodegenerative diseases, the most common disorder of which is Alzheimer’s disease (AD).

Major pathognomonic signatures of rare familial AD (FAD) and the majority of late-onset sporadic forms of AD (SAD) are the accumulation of neurotoxic proteins amyloid β (Aβ) and hyperphosphorylated Tau and cognitive decline with the loss of memory^21–23^. Mutations in FAD have been identified in the amyloid precursor protein (APP), presenilin 1 (PSEN1), and presenilin 2 (PSEN2) genes. Perturbation of the non-amyloidogenic processing pathway of APP causes the accumulation of mainly neurotoxic Aβ in FAD and the majority of SAD. Although comprehensive insight into the processing has been elaborated from several genetically engineered mouse models^24^, upstream molecular pathogenic mechanisms, leading to neurotoxic end products, are still enigmatic, and the validity of the pathognomonic “amyloid cascade hypothesis”^21,25^ has been challenged by several caveats^26,27^. As a first step in understanding the pathogenesis of AD risk, genome-wide associated studies and exome sequencing have identified polymorphisms and coding variants of several genes, which are involved in inflammatory response, endocytosis, and lipid metabolism. As well as the apoEε4 allele^28,29^, the neuronal apoE-receptor, apolipoprotein apoJ^30,31^ and recently phospholipase D3^32^ have been recognized as strong risk factors associated with late-onset SAD^33^. The role of sphingolipids in neurodegeneration has received increasing attention recently, for review see^34^.

In this study, we describe a molecular pathogenic link between SMPD3 deficiency in the neuronal Golgi complex, perturbed secretory pathway and dysproteostasis, thereby eliciting cytotoxicity and AD-like cognitive deficiencies. These data provide a direct neuron-based pathogenetic pathway connecting the perturbation of the Golgi lipid bilayer structure and neurodegeneration in the SMPD3-deficient mouse brain. This phenotype suggests the implication of *smpd3* as susceptibility gene of AD.

## Results

### Neutral sphingomyelinase (SMPD3) expression in CNS is restricted to the Golgi complex of neurons

Multi-tissue expression pattern disclosed the most abundant expression of *smpd3* in brain^14^. Studies on the subcellular localization revealed SMPD3 as key enzyme in the SM cycle of the Golgi compartment (GC) of extra-neuronal tissues^18^.

We first assessed the cell type specific expression of *smpd3* in brain, and the subcellular localization of SMPD3. Neurons, oligodendrocytes and astrocytes of p4-mice were isolated by magnetic cell separation (MACS) from brain homogenates and grown in culture.

**Fig. 1:**
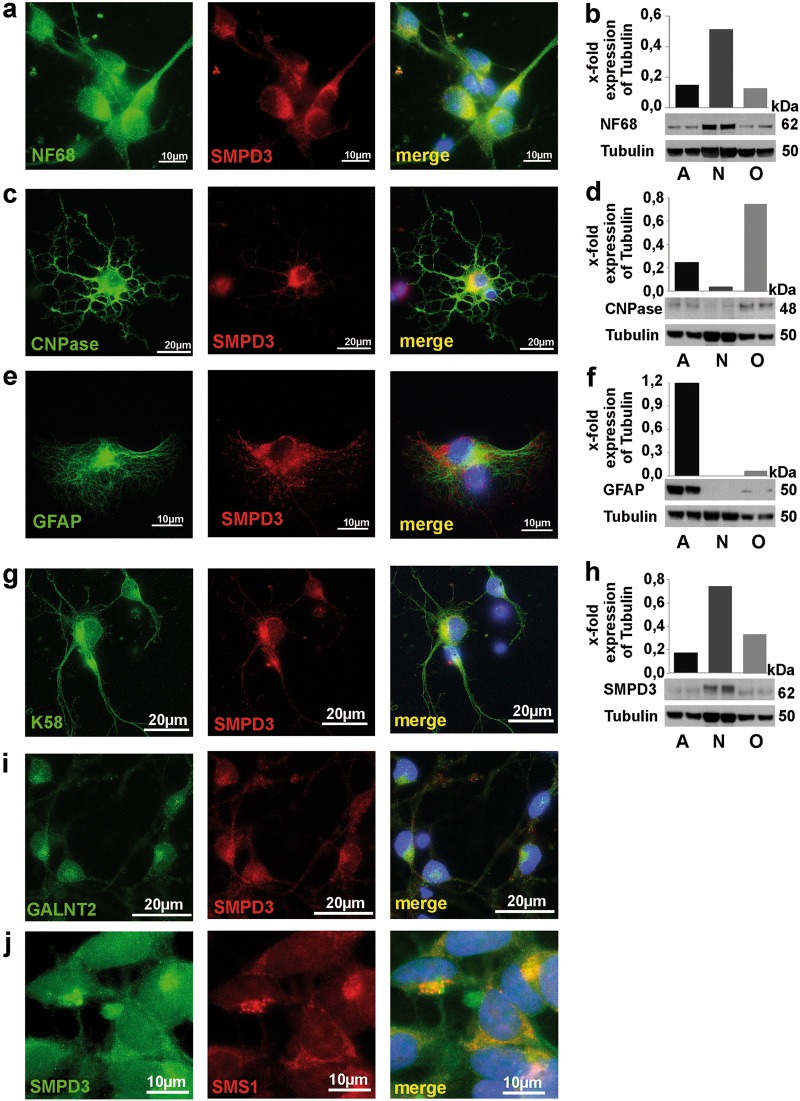
SMPD3 expression in CNS is most abundant to neurons. Immuno-magnetic cell separation and characterization by IHC of **a** neurons of control C57BL/6 mouse brain (p4), labeled with anti-SMPD3 and NF68 antibodies, **c** oligodendrocytes, labeled with anti-SMPD3 and anti-CNPase antibodies, **e** astrocytes, double labeled with anti-SMPD3 and anti-GFAP antibodies. Western blot of isolated cell types, using cell-specific antibodies: **b** NF68, **d** CNPase, **f** GFAP, and **h** SMPD3 in astrocytes (A), neurons (N), and oligodendrocytes (O). Colocalization of **g** SMPD3 and K58 and of **i** SMPD3 and GALNT2 in Golgi complex of neurons. **j** Colocalization of SMPD3 and SMS1 in Golgi of HEK293 cells

Immunohistochemistry (IHC) detected SMPD3 most abundantly in neurons, isolated by depletion of non-neuronal cells from total brain cell homogenate as described under Materials and Methods, characterized by NF68 (Fig. 1a). Minor expression in oligodendrocytes, separated by antiO4-beads and characterized by immuno-labeling with anti- 2′,3′-cyclic-nucleotide 3′-phosphodiesterase (CNPase) antibody (Fig. 1c), and astrocytes, separated by anti-ACSA-2-tagged beads and identified by glial-fibrillary acidic protein (GFAP) (Fig. 1e).

Western blots underscore the prevalent expression of SMPD3 in neurons as well as the purity of the isolated cell types (Fig. 1b, d, f, h).

**Fig. 2:**
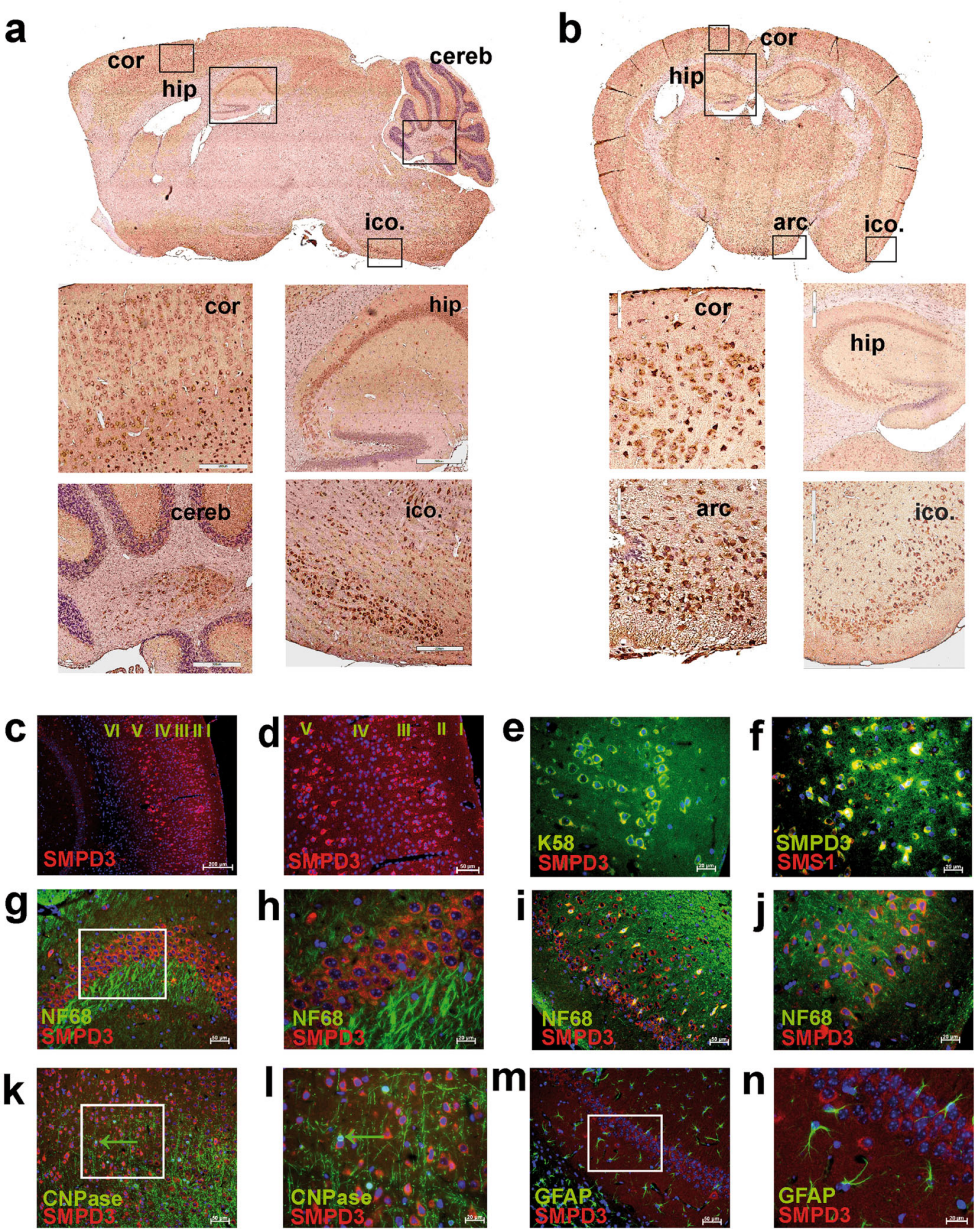
SMPD3 expression in CNS is restricted to neurons predominantly of cortex, hippocampus and hypothalamus. **a** Sagittal and **b** coronal sections of adult brains (6 mo) IHC-labeled with anti-SMPD3 and HRP-tagged secondary antibody. Encased areas of cortex (cor), cerebellum (cereb), hippocampus (hip), inner cortex (ico), and N. arcuatus (arc) are magnified. **c**, **d** SMPD3 expression in neurons of cortical layers III and V. Colocalization of **e** SMPD3 and K58 and **f** SMPD3 and SMS1 in Golgi complex of neurons. Merged images of **g**, **h** hippocampus and **i**, **j** cortical sections, labeled with anti-SMPD3 and neuron-specific NF68 antibodies. **k**, **l** Minor expression of SMPD3 in CNPase-labeled oligodendrocytes (arrows), and **m**, **n** GFAP-labeled astrocytes

High-resolution microscopy of immune-stained neurons colocalizes SMPD3 with Golgi-marker K58 and GALNT2 in the Golgi complex (Fig. 1g, i) and with SMS1 in Golgi of HEK293 cells (Fig. 1j) in addition to coronal sections of cortex (Fig. 2f).

We mapped SMPD3 expression in sagittal (Fig. 2a) and coronal sections (Fig. 2b) of adult Cntr brains (6 mo) immuno-histochemically, using anti-SMPD3 and horseradish peroxidase-tagged secondary antibody. Neurons of cortical layers III and V, of subiculum and CA1–CA3 regions of hippocampus, and of hypothalamus showed highest SMPD3 expression.

Cortical external pyramidal cell layer III and internal ganglionic cell layer V, hippocampus, dentate gyrus and nuclei of the hypothalamus of adult brain (6 mo) showed high SMPD3 expression (Fig. 2c, d). Neurons, oligodendrocytes, and astrocytes were also identified in adult control (Cntr) mouse brain by double labeling with anti-SMPD3 and NF68, anti-CNPase and anti-GFAP antibodies, respectively (Fig. 2g–n). These studies *in vivo* and *in vitro* allocated SMPD3 expression predominantly to neurons. High-resolution confocal microscopy colocalized SMPD3 with Golgi-marker K58 and with SMS1 in the neuronal Golgi complex (Fig. 2e, f and Supplementary Fig. 1a).

### Smpd3 deficiency causes accumulation of APP, Aβ, and pTau in neurons

**Fig. 3:**
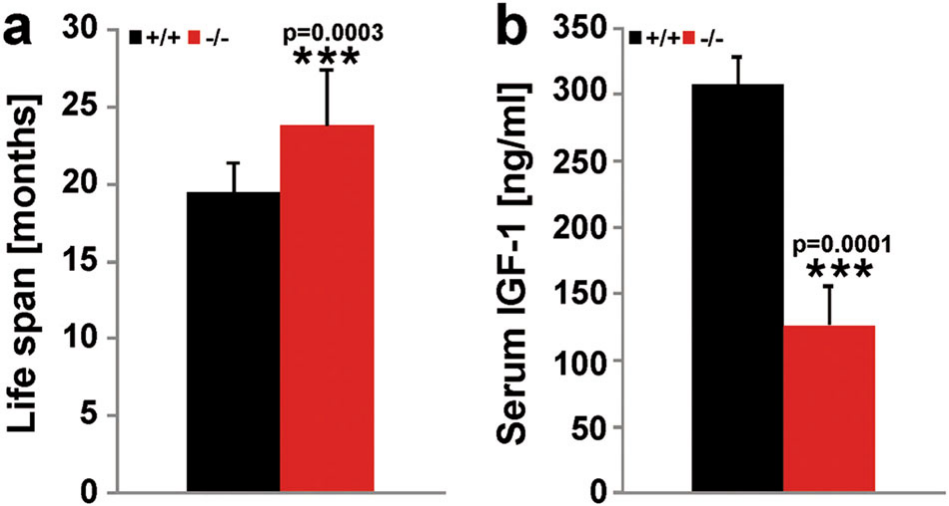
Longevity and reduced IGF-1 serum levels of SMPD3-deficient mice. (**a** Lifespan of Cntr and *smpd3-/-* mice (*n* = 20). **b** IGF-1 serum levels of Cntr and *smpd3-/-* mice (*n* = 10). *p* values of ≤0.05 *, ≤0.01 **, ≤0.001 *** were considered significant

SMPD3 deficiency in hypothalamic neurosecretory neurons perturbs the GHRH/GH/Igf1-axis^17^. A significantly expanded lifespan (up to 20%) (Fig. 3a) is caused by postnatal and juvenile Igf1- serum levels, which remain constantly low during adulthood (Fig. 3b)^35,36^.

**Fig. 4:**
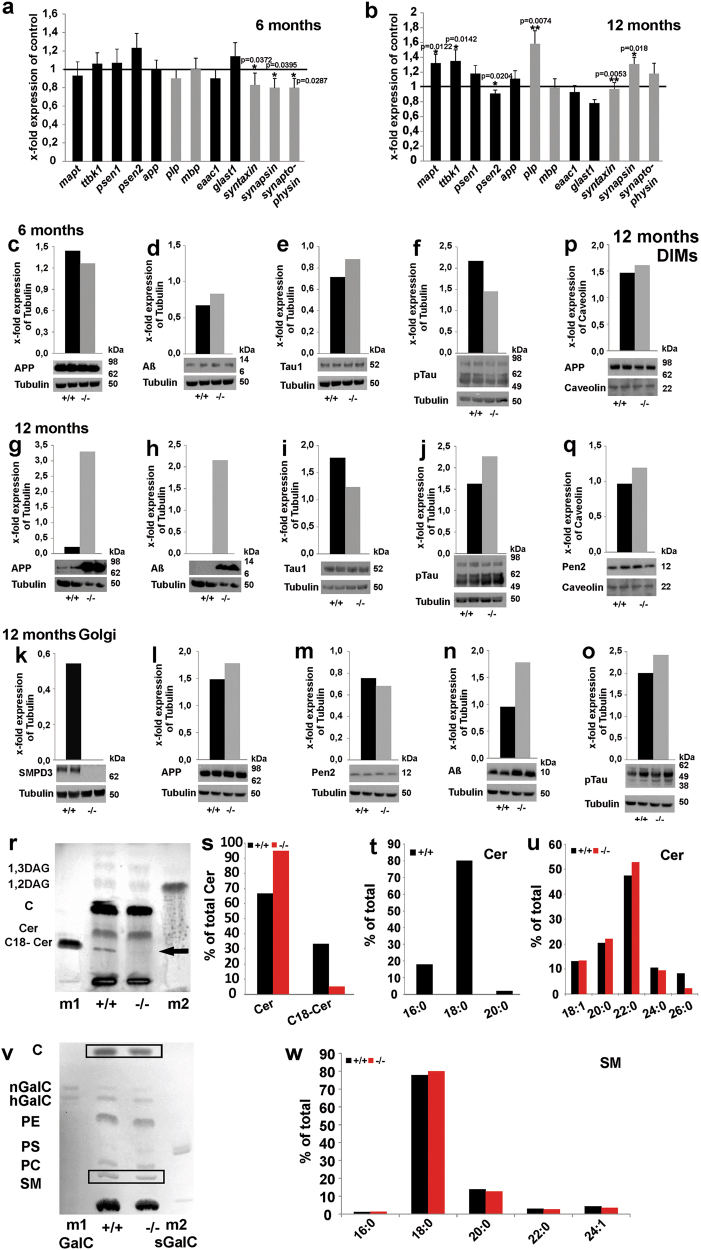
Quantitative analysis of gene- and protein-expression and lipidomes of Cntr and *smpd3-/-* Golgi complexes. Real-time-PCR of total brain RNA of cohorts (*n* = 5) of **a** 6- and **b** 12-mo-old Cntr and *smpd3-/-* mice, using neuron-specific markers *syntaxin, synapsin,* and *synaptophysin*, of AD-related genes *mapt*, *ttbk1, app*, and *psen1* and *2*; oligodendrocyte-specific markers *plp* and *mbp*; and astrocytes-specific markers *eaac1* and *glast1*, mean ± SD. Representative WB of lysates of **c–f** 6 and **g**–**j** 12-mo-old total brain using anti -APP, -Aβ, -Tau1, and -pTau, of **k–o**. Golgi complex using anti –SMPD3, -APP, -Pen2, -Aβ, and -pTau of **p**, **q** Golgi DIMs, using anti-APP and -Pen2 antibodies of Cntr and SMPD3-/- mice (*n* = 3). **r** HPTLC of Cer and DAG pools of the Golgi lipid extract. Markers: m1 18:0-Cer, m2 1,2-18:1/18:1-DAG **s** Quantification of Cer species, **t** “C18-Cer” species (arrow in **r**), **u** identical ceramide-species in >20-ceramides. **v** HPTLC of Golgi lipids. **w** MS/MS-analysis of SM species. C cholesterol, CMH ceramide monohexoside, PE phosphatidyl ethanolamine, PS phosphatidyl serine, PC phosphatidyl choline, SM sphingomyelin

We investigated age-dependent gene expression in brain by real-time PCR, using primer pairs (Table S1), of neuron-specific *syntaxin, synapsin* and *synaptophysin*, oligodendrocyte-specific *plp* (*proteolipidprotein*), astrocyte-specific *glast1 (glutamate-aspartate transporter 1)* and *eaac1 (excitatory amino acid carrier 1)* and AD-related *app* (*amyloid precursor protein*), *mapt* (*microtubule associated tau potein*), *ttbk1* (*tau tubulin kinase1*), and *psen 1* and *2* (*presenilin*) in 6- and 12-mo-old Cntr and *smpd3-/-* mice. *Smpd3-/-* mice, at age 12 mo, showed significantly elevated steady-state mRNA levels of the neuron-specific marker genes *mapt* and *ttbk*, and likewise of *plp*, whereas *app* expression remained unchanged (Fig. 4a, b).

We isolated the Golgi fraction of brain of 12-mo-old Cntr and *smpd3-/-* mice and their detergent-insoluble membrane (DIM) domains for western blot hybridization and lipidomic analyses. The purity of the Golgi fraction was confirmed by western blot analyses probing the Golgi complex by anti-K58, and plasma membrane contaminants by the anti-Na+/K+-ATPase antibody (Supplementary Fig. 1b, c). Western blot hybridization analysis of protein lysates of mutant Golgi proved the absence of SMPD3 (Fig. 2k). WB of total brain, the Golgi-, and its DIM-fraction of Cntr and *smpd3-/-* mice revealed the association of APP, Aβ, and of Pen2, the catalytic subunit of γ-secretase with the Golgi-DIM-fraction, (Fig. 4c–o). Concentrations of APP and its amyloidogenic Aβ considerably increased in brain of *smpd3-/-* mice between age 6 mo (Fig. 4c, d) and 12 mo (Fig. 4g, h). Aβ of Golgi fraction in smpd3-/- mice of 12 mo is increased in comparison with Cntr (Fig. 4n). Similar to the elevated steady-state, RNA concentration of neuron-specific markers *mapt* and *ttbk* (Fig. 4a, b), pTau protein concentration in the lysate of total brain of *smpd3-/-* mice significantly increased between 6 and 12 mo of age (Fig. 4f, j) and is elevated in Golgi fraction of 12-mo-old smpd3-/- mice in comparison with Cntr (Fig. 4o). APP and Pen2 concentrations remained unchanged in Golgi and DIMs of Golgi of 12-mo-old Cntr and *smpd3-/-* mice (Fig. 4l, m, p, q).

### SMPD3 deficiency in the neuronal GC disrupts remodeling of the lipid bilayer essential for the Golgi secretory pathway

SM/cholesterol-enriched DIM domains are embedded in the phospholipid-rich domains of the luminal leaflet of Golgi membranes^37^. DIMs of the Golgi complex of Cntr neurons are the scaffold of SMPD3 and SMS1^18^. High-performance thin-layer chromatography (HPTLC)- MS/MS species analysis of the SM- and Cer-pools of Golgi membranes of Cntr and *smpd3-/-* mice revealed that >80% of SM-pool consisted of the C18-SM species (Fig. 4w and Supplementary Fig. 2d, e). Only the Golgi Cer-pool of Cntr neurons contained the distinct C18-Cer fraction (Fig. 4r), which is absent in SMPD3-deficient Golgi membranes (Fig. 4r–t). Absence of SMPD3 in the Golgi–SM cycle leads to restricted production of C18-Cer, which is the acceptor substrate of the SMS1-catalyzed group transfer of phosphoryl-choline from phosphatidyl choline, and of DAG (Fig. 4r–w).

Combined HPTLC- MS/MS of the Golgi DAG pool of Cntr mice revealed a a cluster of DAG species, reflecting the backbone of Golgi-PC, and another with the major species 18:0/20:4-DAG, released by specific phospholipase C from phosphatidyl inositol-phosphates in the plasma membrane and ER (Supplementary Fig. 2a–c).

Cntr and *smpd3-/-* Golgi contained similar pattern and equal concentrations of very long chain (C20-C26)-Cer, serving as substrates of UDP-glycosyl-transferase for mono- and poly-hexosyl-ceramide synthesis (Fig. 4u–w). The phospholipidomes of the Golgi fraction of Cntr and *smpd3-/-* brain were similar (Fig. 4r–t, Supplementary Fig. 1d–i).

### Inhibition of APP, Aβ, and pTau- protein transport, perturbation Golgi secretory pathway and apoptosis in SMPD3-deficient neurons

**Fig. 5:**
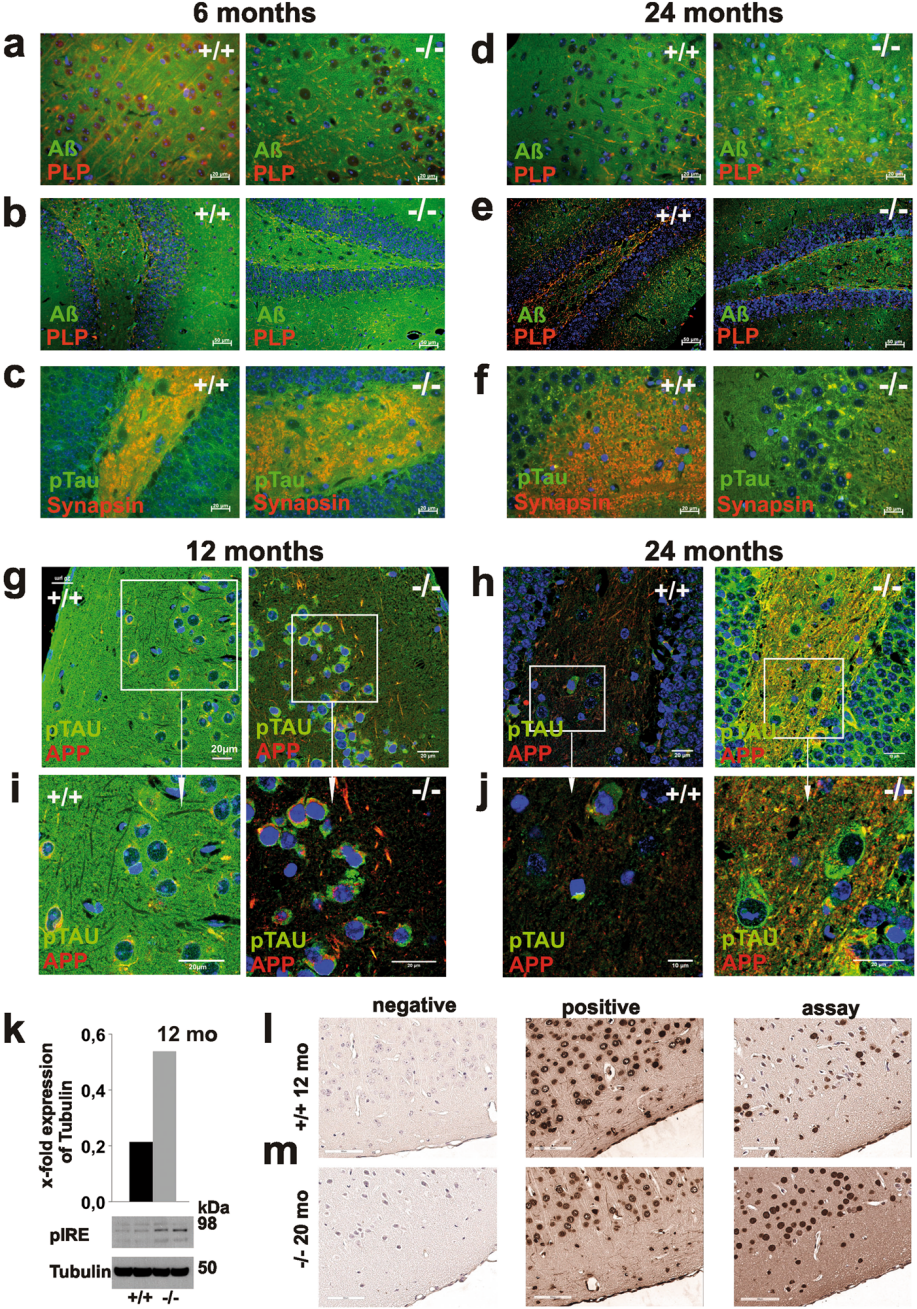
Age-dependent neuronal dysproteostasis in *smpd3-/-* mice. Merged epifluorescence images of brain sections of 6- and 24-mo-old Cntr and *smpd3-/-* mice revealed age-dependent heavy accumulation of Aβ and pTau in neurons of *smpd3-/-* mice with age, Aβ in **a**, **d** cortex and **b**, **e** dentate gyrus and **c**, **f** pTau in dentate gyrus and hippocampus, using anti-Aβ/anti-PLP and anti-pTau/anti-Synapsin-antibodies. Confocal images of **g–j** cortex and dentate gyrus of 12- and 24-mo-old Cntr and *smpd3-/-* brain sections double labeled with anti-APP and anti-pTau (AT8) antibody, **i**, **j** enlarged images of encased areas. **k** Representative WB of pIRE in 12-mo-old *smpd3-/-* mice (*n* = 4). **l**, **m** TUNEL assay in section of cortex of 12- and 20-mo-old Cntr and *smpd3-/-* mouse brains, counter-stained with haemalum

The marked increase of APP, Aβ, and pTau in western blot hybridization analysis of 12 mo of age *smpd3-/-* brain lysates (Fig. 4g, h, j) became also apparent in IHC (Fig. 5a–j). Anti-Aβ-antibodies displayed Aβ-deposition in neurons of cortex of 6-mo-old *smpd3-/-* mice (Fig. 5a), heavy Aβ-loaded Golgi of cortical neurons (Fig. 5d) and neurons of dentate gyrus (Fig. 5e) of *smpd3-/-* mice 24 mo of age. APP and intra- and extracellular pTau deposits were observed in neurons of the cortex already in *smpd3-/-* mice 12 mo of age (Fig. 5g–j). We addressed the impaired proteostasis by quantitative western blot analysis of unfolded protein response using marker pIRE. Elevated pIRE expression strongly indicated the activation of endoplasmatic reticulum stress signaling pathways (Fig. 5k). TUNEL staining was strongly increased in coronal and sagittal brain sections of 12-mo-old *smpd3-/*- compared with Cntr mice (Fig. 5l, m). IHC of coronal brain sections of *smpd3-/-* mice revealed an age-dependent increased number of AnnexinV-positive neurons, (Fig. 5n, o).

### **Impaired Golgi secretory pathway in*smpd3-/-*neurons triggers progressive AD-like cognitive decline**

**Fig. 6:**
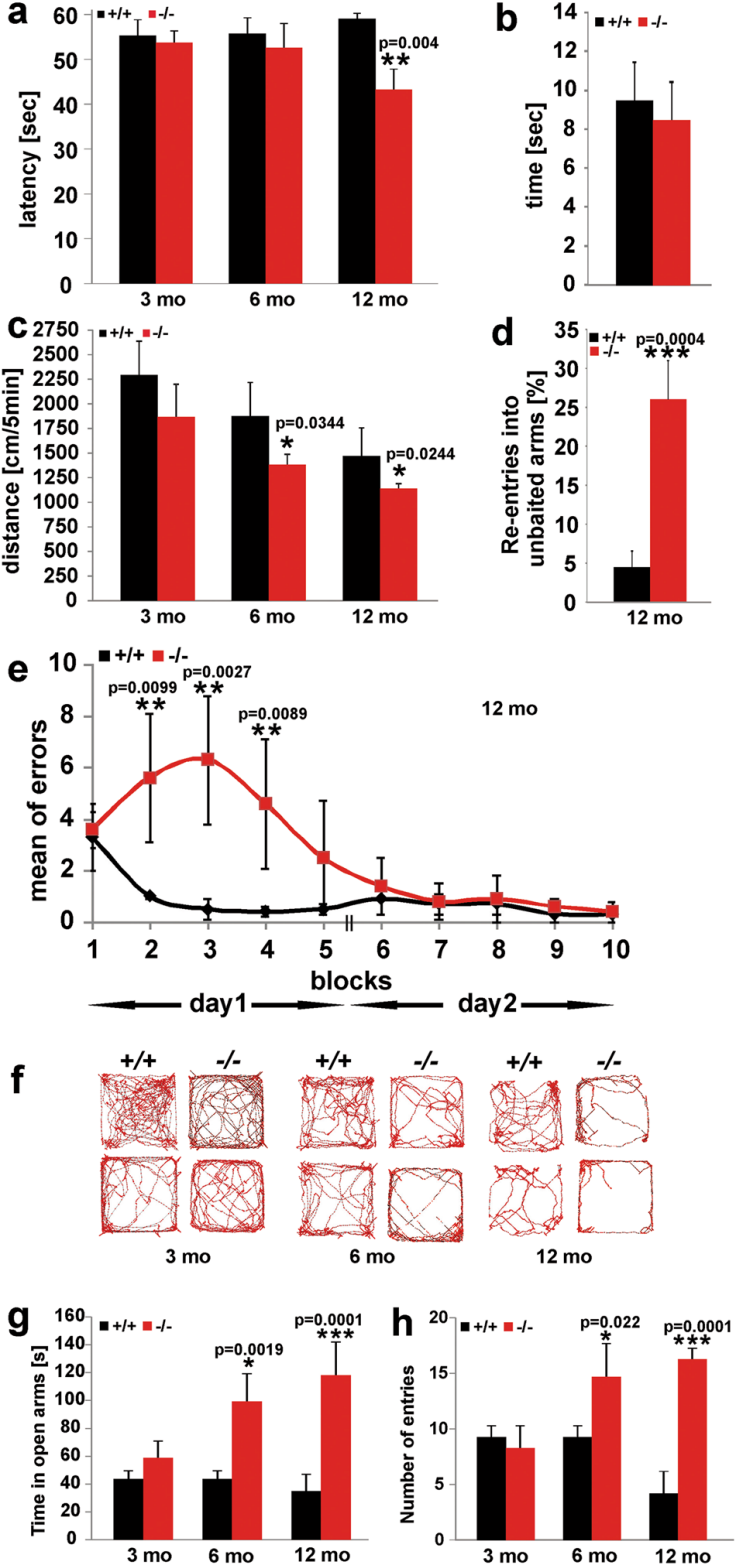
Age-dependent decline of motor activity, coordination, and cognitive tasks of SMPD3-deficient mice. Assessment of motor activity and coordination by **a** Rota-rod task, **b** beam walk task, and **c** open field test, working memory by **d** T-maze, reference memory by **e** RAWM of Cntr and *smpd3-/-* mice, age 12 mo, **f** thigmotaxis in open field task and anxiety response by **g**, **h** elevated plus-maze. *N* = 8, mean ± SD. *p* values of ≤ 0.05 *, ≤ 0.01 **, ≤ 0.001 *** were considered significant

We next tested the age-dependent performance of cohorts of gender and weight matched Cntr and *smpd3-/-* mice, age 3, 6 and 12 mo in a battery of tasks, motor activity, and coordination in the Rota-rod- and beam walk and locomotor activity I the open field test. Despite skeletal deformations of the *smpd3-/-* mutant^17^, their performance in the Rota-rod test matched that of Cntr cohorts up to age 6 mo, but significantly declined at age 12 mo (Fig. 6a). Performance in the beam walk task remained unchanged (Fig. 6b). Open field ambulatory movement (distance run/5 min) was reduced in 3-mo-old *smpd3-/-* mice, and further decreased by 25% at age 6 mo and 25% at age 12 mo, similar to Cntr mice (Fig. 6c).

Cognitive defects were explored by running a behavioral test battery^38,39^ with two groups of 12-mo-old Cntr and *smpd3-/-* mice. The performance of *smpd3-/-* mice in the T-maze at age 12 mo showed severe deficits in the working memory (Fig. 6d), and in the radial arm water maze (RAWM) the impaired reference memory task (Fig. 6e).

Explorative motivation was measured in the open field task with the video-tracking technique, (Fig. 6f). Unlike the Cntr cohorts, 6- and 12-mo-old *smpd3-/-* mice barely overcame the initial thigmotaxis phase to enter the second phase of exploring the environment for cues of spatial representation. Furthermore, we assessed the anxiety-related behavior of Cntr and *smpd3-/-* mice, age 3, 6, and 12 mo, in the elevated plus-maze. *smpd3-/-* mice developed a strongly reduced anxiogenic phenotype with age (Fig. 6g, h), indicated by loss of proclivity toward dark and loss of avoidance of open space and height. *smpd3-/-* mice, age 6 mo spent twice and age 12 mo and four times longer time-intervals in the open arm and entered the closed, safe arm four times more frequently than Cntr mice.

## Discussion

Here, we describe a novel pathogenetic process involving neuronal SMPD3 deficiency in CNS of the *smpd3-/-* mouse mutant. We unveiled a molecular link between SMPD3 deficiency in the SM cycle of the neuronal Golgi complex, impeded remodeling of the lipid bilayer of the Golgi membrane, essential for budding, vesicle formation and protein transport of APP, Aβ and pTau, dysproteostasis, causing neurodegeneration and AD—like cognitive decline.

Our mechanistic studies demanded the unambiguous assignment of the cellular and subcellular localization of SMPD3 in CNS. IHC of isolated neurons, oligodendrocytes, and astrocytes of p4-mouse brain, using anti-SMPD3 antibodies and cell-specific markers convincingly demonstrated that anti-NF68 positive neurons are the main scaffold of brain SMPD3, minor expression is found in anti-CNPase-positive oligodendrocytes and anti-GFAP-positive astrocytes. IHC-mapping of neuronal SMPD3 expression in sagittal and coronal sections of brain uncovered strongest expression of SMPD3 in neurons in cortical layers III and V, of CA1 and III of hippocampus and dentate gyrus, cerebellum, and hypothalamus.

Cell fractionation, biochemical, and immuno-histochemical studies identified DIMs of the neuronal GC as the subcellular platform for the concerted action of integral polytopic SMPD3 and SMS1 and their substrates and products SM, Cer, PC, and DAG in the SM cycle. SM and cholesterol are major constituents segregated into DIM domains, lo—(liquid ordered) DIMS of the Golgi lipid bilayer and embedded in ld (liquid disordered) domains (van Meer et al. 2008). Sub-fractionation of the Golgi membrane stacks of peripheral tissue allocated SMPD3 and SMS1 to DIM domains^18^. The association of SMS1 with the trans Golgi network has been reported earlier^40^.

Lipidomic analysis of the GC-membrane lipid bilayer revealed that the Golgi–SM fraction consists >80% of the stearoyl-SM species (C18-SM), asymmetrically distributed in the luminal leaflet of the Golgi lipid bilayer, and two pools of Cer species in Cntr Golgi, a distinct C18-Cer fraction and a long chain Cer fraction. C18-Cer reflects the backbone of Golgi-C18-SM. This C18-Cer fraction is absent in SMPD3-deficient Golgi membranes. A second pool consists of very long chain (>C20) *de novo* synthesized ceramides (Cer) in Cntr and *smpd3-/-* Golgi complexes only being utilized for glycosphingolipid synthesis.

These observations suggest a central role of C18-SM and C18-Cer in the SM cycle confined to DIMS of Golgi membranes.

The domain structure compartmentalizes cellular processes. SMPD3 and SMS1 are embedded in the C18-SM-enriched lipid bilayer of DIMS, the platform of the Golgi–SM cycle.

**Fig. 7:**
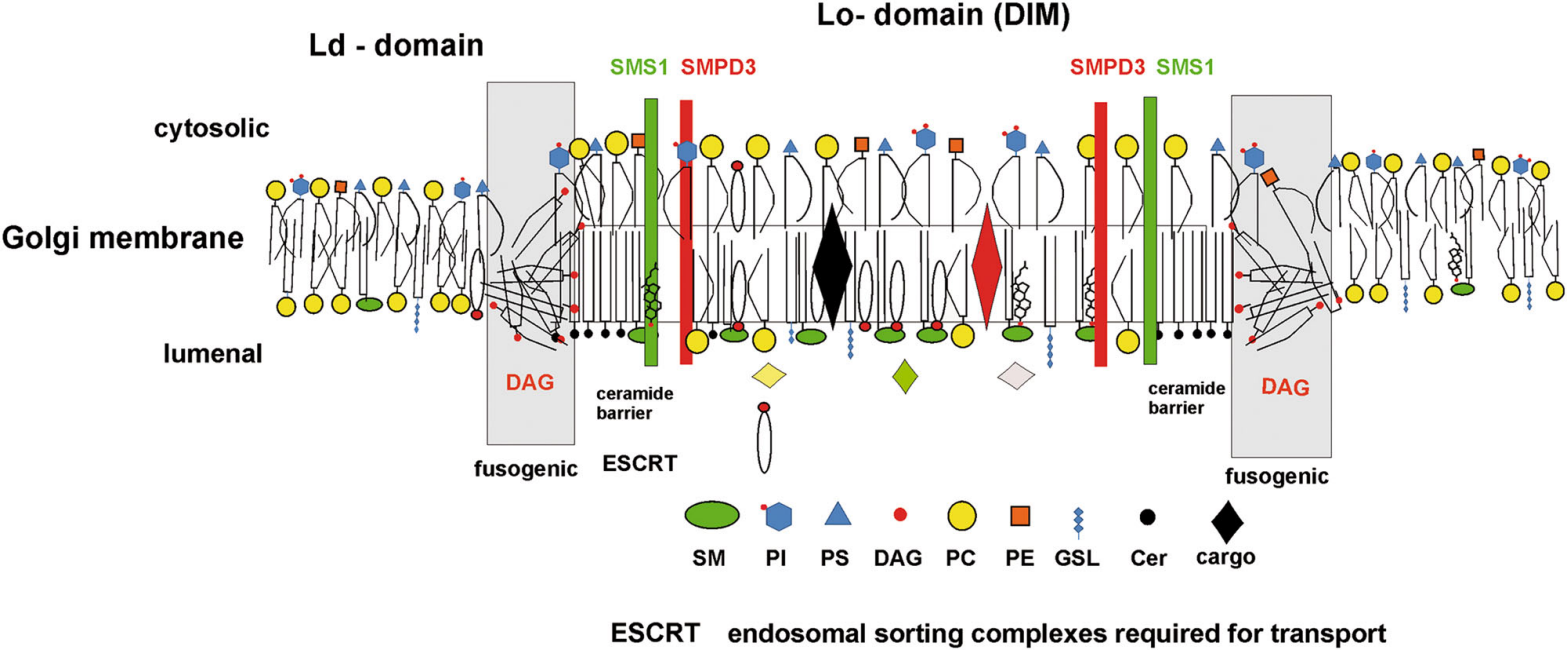
Suggested mechanistic model of lipid-driven bilayer remodeling in bud formation. C cholesterol, SM sphingomyelin, PI phosphatidyl inositol, PS phosphatidyl serine, DAG diacyl glycerol, PC phosphatidyl choline, PE phosphatidyl ethanolamine, GSL glycosphingolipid, Cer ceramide, ESCRT endosomal sorting complexes required for transport

SMPD3-hydrolysis of C18-SM in the Golgi luminal leaflet triggers a reaction-cascade: the polar head group of SM is detached by SMPD3, thereby removing the “umbrella” effect of SM in the SM/C complex with the dissociation of C18-ceramide, which aggregates owing to its physical properties to a lamellar Cer-barrier boarding the lo-DIM-domain in the luminal leaflet, Fig. 7.

This concept is in agreement with kinetic studies in model systems consisting of phase-separated ternary SM/PC/C domains embedded in a liquid disordered PC lipid bilayer, when treated with bacterial sphingomyelinase^41,42^.

C18-SM has been observed to bind to a specific amino acid sequence (signature) in the transmembrane domain of p24, a subunit of the COPI complex of COPI vesicles, the anterograde Golgi-ER-carriers, which are recruited in the Ld-domain^43^. Remarkably, this C18-SM signature sequence is also present in the Psen2-subunit of γ-secretase, and in Aβ-protein^44^.

We located Pen2 and APP in DIMs of the Golgi membranes of Cntr neurons. Palmitoylated beta-secretase1 (beta-site amyloid precursor protein cleaving enzyme 1), which initiates the amyloidogenic pathway and the γ-secretase tetrameric complex of PSN1, nicastrin, APH1, and Pen2 are also segregated to lo- domains for APP processing (Kitazume et al. 2001)^45^. Absence of SMPD3 in *smpd3-/-* Golgi DIMS leads to partitioning of SMS1 between lo- and ld-domains^18^.

SMS1-catalyzed group transfer of phosphoryl-choline from phosphatidyl choline to Cer releases DAG. Unlike Cer, DAG has membrane fusogenic properties lipid, freely equilibrates between the bilayer, disintegrating locally the bilayer structure. In addition the molecular shape of DAG imposes a large negative curvature upon the membrane, which facilitates constriction of the membrane into a neck-structure^46^ and triggers budding initiating vesicle formation and cargo transport in the Golgi secretory pathway. Whether this lipid-driven bud formation requires ESCRT (endosome sorting complex required for transport) remains to be answered.

SMPD3 deficiency prohibits the “shuttle” function of Cer in the dynamic modulations of the Golgi lipid bilayer for budding, a fundamental process in neuronal protein transport, documented in the age-dependent accumulation of APP, Aβ and pTau in neurons of cortex, hippocampus, dentate gyrus and hypothalamus of 6- and 12-mo-old *SMPD3-/-* mice. Impeded proteostasis and accumulation of neurotoxic end products in neurons activated ER- stress signaling. Elevated expression of pIRE in western blot based quantitative analysis and TUNEL assay of coronal brain sections of *smpd3-/-* mice (12 mo) assessed increased age-dependent apoptosis of *smpd3-/*- neurons.

Studies on the sorting of membrane into vesicles of multivesicular bodies in PLP-egfp-overexpressing oligodendrocytes transfected with nSMase2 and in giant vesicles I experiments *in vitro* when treated with bacterial SMase released the ceramide^47^. Ceramides were held responsible for the transfer of exosome-associated domains into the lumen of the endosome in an ESCRT independent manner.

Motor activity and coordination of SMPD3-deficient and Cntr mice were evaluated in the Rota-rod and horizontal beam walk crossing tasks. Performance in the Rota-rod task indicated impaired motor activity and coordination in male and female *smpd3-/-* mice compared with Cntr mice at age 12 mo, but similar performance in the beam walk task.

Cognitive defects were explored by running a behavioral test battery^38,39^ with two groups of gender and weight matched 3, 6- and 12-mo-old Cntr and *smpd3-/-* mice.

Working and reference memory were probed in the T- maze and the RAWM. *smpd3-/-* mice developed severe deficits in the working memory- and reference memory tasks at age 12 mo. T- maze and RAWM have proven to be sensitive and reliable tasks in uncovering the moderately impaired cognitive functions of AD mouse models^48^ associated with dysfunction of hippocampus, and in detecting memory deficits in APP transgenic mouse mutants^49,50^.

Explorative motivation was measured in the open field task. Unlike the 6- and 12-mo-old Cntr cohorts, *smpd3-/-* mice barely overcame the initial thigmotaxis phase to enter the second phase, exploring the environment for cues of spatial representation.

*Smpd3-/-* mice developed a strongly reduced anxiogenic phenotype with age, as indicated in the elevated plus-maze by loss of proclivity toward dark and loss of avoidance of open space and height.

A recent study^51^ to assess the function of SMPD3 in AD-related pathology, using a *5xfad;fro-/-* double mutant mouse^52^, is incompatible with the study reported here, using the unbiased *smpd3-/-* mutant, as substantiated by the high postnatal lethality of the *fro-/-* mutant compared with the extended lifespan (>20%) of the *smpd3*-/- mouse mutant and by the contrasting skeletal phenotypes of the *fro-/-* and *smpd3-/-* mutants^19^.

The low-serum insulin-like growth factor 1 concentration in juvenile smpd3 mice^17^ persisted in adult smpd3 mice and resulted in an extended lifespan (>20%), which is owing to the hypothylamic pituitary growth axis^53,54^.

Our study provides a novel concept of the pathogenic basis of the nexus between *smpd3*-gene ablation, which perturbs the SM metabolism in the neuronal GC and the lipid bilayer modification essential for lipid-driven budding and vesicle formation, and triggers dysproteostasis, apoptosis, and neurodegeneration. Further studies are needed to support the suggested function of *smpd3* as Alzheimer´s disease susceptibility gene.

We anticipate this discovery might contribute to the search for other primary pathogenic mechanisms in remodeling the lipid bilayer of neuronal Golgi membranes essential for protein processing and transport triggering age-dependent neurodegeneration and cognitive deficits.

## Materials and Methods

### Mouse lines

The *smpd3-/-* mouse line was developed in this laboratory, and after 10 back-crossings maintained on a C57Bl/6 background^17^. Control (Cntr) mice were obtained from heterozygous *SMPD3-/-*×C57Bl/6 crossings. Mice were genotyped by PCR analysis of tail DNA. Cohorts of gender, age and weight matched Cntr and *smpd3-/-* mice were used in this study. Animals were kept under pathogen-free conditions. The ARRIVE Guidelines^55^ have been followed in the animal studies reported in this manuscript.

### Dissociated neuronal cultures

Brains of p5 pups of Cntr mice were dissociated to single-cell suspensions using the Neural Tissue Dissociation Kit, Miltenyi Biotec, following the manufacturer’s protocol. Mouse neurons were isolated by depletion of non-neuronal cells, following the manufacturer’s protocol. Non-neuronal cells were magnetically labeled with biotin-conjugated monoclonal antibodies specific for non-neuronal cells to be retained by anti-biotin monoclonal antibodies conjugated MicroBeads packed into MACS Column. Unlabeled neuronal cells were collected in the flow-through and cultured in six well Costar-plates.

Astrocytes were isolated using the anti-ACSA-2 (astrocyte cell surface antigen-2) MicroBead Kit, Miltenyi Biotec. Fc receptors were blocked with FcR blocking reagent and then the ACSA-2+ cells were magnetically labeled with anti-ACSA-2 and separated according to the manufacturer’s instructions.

### Real-time PCR

RNA from Cntr and *smpd3-/-* brains of littermates (*n* = 5) was isolated using Trizol, Invitrogen. In total, 10 µg of total RNA was reverse-transcribed using a transcriptase kit, life technologies^18^. Primer pairs used in quantitative PCR reactions are listed in Table S1. *Hgprt* was used as internal standard. Quantitative PCR reactions were performed with the ABI Prism 7900HT employing a 96well format and the Fast SYBR Green Master Mix, Applied Biosystems, following the manufacturer´s protocol. Data analysis was performed using the 2-ΔΔCt method.

### Acid and neutral sphingomyelinase assays

Radioactive assay for acid and neutral sphingomyelinase activity was performed as described previously^5,13^.

### Cell fractionation

Cell fractionation, isolation of Golgi fractions^56^, and Triton X-100 insoluble DIMs^57^ from Golgi fractions were performed following established procedures.

### Histology and IHC

Cntr and *smpd3-/-* mice, 6 and 24 mo of age, were perfused from the left ventricle with 25 ml phosphate-buffered saline (PBS) and with 50 ml PBS-buffered 4% paraformaldehyde for cryo- and paraffin embedding and processing for light‐ and immunofluorescence microscopy. Coronal and sagittal sections (5 µm) were permeabilized with 0.5% Triton X-100/PBS at 4 °C, blocked with 3% bovine serum albumin /PBS and treated with respective antibody dilutions in tris-buffered saline, supplemented with 5% non-fat dry milk at 4 °C over-night. The following antibodies and dilutions were used: affinity purified anti-PLP, 1:100^58^; anti-SMPD3, 1:200,^14^; anti-Amyloid 1:500 clone WO2, Merk Millipore, MABN10; anti-CNP, 1:250, abcam #ab6319; anti-Synapsin I, 1:300, abcam, #ab64581; anti-Tau1 1:100. Anti-pTau, 1:500, ThermoFisher Scientific, #MN1020; anti-GFAP, 1:400, Sigma-Aldrich, #G3893, anti-NF68, 1:300, Sigma-Aldrich, #N5139.

Sections were washed with PBS/0,5% Triton X-100, incubated with Cy3-conjugated second IgG antibody (Jackson Immuno Research) for 1 h at 37 °C, washed with PBS/0.5% Triton X-100 and stained with hematoxylin–eosin for transmission microscopy or immuno-stained with affinity purified rabbit polyclonal or monoclonal antibodies. A Zeiss microscope Axio ImagerM1 and the AxioVision Imaging Software were used for light- and fluorescence microscopy The TCS SP8X confocal microscope (Leica Microsystems), equipped with a PL Apo 63 × /1.40 Oil CS2 objective, white light laser (NKT Photonics) and HyD detectors was used for confocal microscopy.

### Apoptosis assays

Neuronal apoptosis was visualized using the TUNEL-Apoptosis Detection Kit #17-141, EMD Millipore, Merck.

### Lipidome analysis

Total lipids were extracted from brain or subcellular fractions for phospholipidomic analysis as outlined before^59^. MS/MS of complex lipids in lipid extracts are described in detail under Supplemental Information.

### Protein analysis by western blotting

Mouse brain proteins were isolated and solubilized for western blot hybridization. Protein aliquots (50–100 µg) were analyzed using the NuPAGE western Blot system (Invitrogen). The following antibodies were used: anti-Amyloid Precursor Protein, 1:20000, abcam, #ab32136; anti-α tubulin, 1:12000, Santa Cruz, #sc5546; anti-Caveolin, 1:5000, BD Transduction Laboratories #610059; anti-pTau, 1:1000, abcam, #ab926767; anti-pIRE, 1:1000, abcam, #ab48187; anti-Pen2, 1:500, abcam, #ab18189, GalNAc-T2, ThermoFisher PA 5-2141. Horseradish peroxidase conjugated secondary antibodies were used for detection with the ECL system. Signals were quantified by densitometry using the IMAGE J2X program.

### Motor activity, behavioral, and cognitive performance

Mice (*n* = 8) were accommodated in home cages under constant lightning, noise levels and temperature 2 h prior to testing. Equipment was cleaned with 70% ethanol between test sessions.

### Motor activity and coordination tests

#### Rotor rod test

Mice were placed on a rotating rod (3.5 cm diameter) 50 cm from the floor in lanes 12 cm wide in the same direction, whereas the bar is rotating (Brooks and Dunnett, 2009. The speed of rotation was set at constant 16 rpm during the 60 s trial. Latencies (seconds) of maintaining balance on the bar were recorded.

### Beam walk maze

The beam walk maze used in this study followed established protocols^60^. In brief, mice were trained to walk along an 80 cm long and 1.2 cm wide wooden beam, bridging two platforms (50 cm above the bench). Mice were placed on the beam and allowed to. The time to full-length traverse the beam and to reach the goal box reach, dropping off the bridge, and the time until the drop off were recorded.

### Open field test

The Open field test was used to assess spontaneous ambulatory movement and spontaneous motor activity as well as the degree of exploring the novel environment resembling anxiety-like behaviors^61^. Distance and speed in ambulatory movement were measured. Mice were placed in an open field maze chamber 50 cm long × 50 cm wide. Activity was recorded using video-tracking software (Stassen, Department of Physiology, University of Cologne). Data were collected continually for five minutes and the distance traveled (cm) and velocity (cm/second), recorded, and scored automatically. Distance traveled and movement speeds assessed ambulatory movement. Thigmotaxis was documented by video-tracking.

### T-maze

The homemade T-maze apparatus was adapted in size to mice, as described before^62^. Mice were food deprived until their body weight attained 85%. They were placed at the base of the T and allowed to choose one of the goal arms. Only one of the arms contained a food reward at the end of the arm. Mice were trained 7–10 days to enter the arm with the food reward, eating the reward and starting again at the base. The food reward was always placed on the side of the maze not entered by the mouse on the previous trial. Spatial working memory was measured by recording the number of correct entries into baited arms and number of re-entries into an unbaited arm. The cutoff time of each trial was 60 s.

### Elevated Plus-Maze

Anxiety-related behavior was assessed by the passive avoidance test. A homemade elevated plus-maze consisting of two enclosed arms and two open arms, elevated 50 cm above the bench was used^63^. To assess anxiety-related behavior, defined as the degree to which the subject avoided the open arms (perceived unsafe arms) of the maze and preferring the closed arms (perceived safe arm) of the maze. Each mouse was placed in the center of the maze The latency and number of entries spent in the preferred closed arms (“safe arm”) and open arms (“unsafe arm”) were recorded over 5 min to define the degree of anxiety-related behavior. One session of 5 min per mouse was carried out.

### RAWM task

In brief, the maze consisted of six arms 160 cm in diameter with arm length 30 cm and circular swim area of 40 cm^50^. The water level of the pool was adjusted to ~ 2 cm above an invisible 10-cm circular platform placed in the back of one arm ~ 7 cm inside the back wall. The pool was located in the center of a dimmed room. Black poster walls with geometric visual cues bordered the pool horse-shoe like.

The RAWM protocol consisted of a 2-day staggered training schedule for cohorts of six mice, alternating through the trials over day 1 and day 2 of the test. During block 1 (six trials) and block 2 (six trials), mice were trained to identify the platform location by alternating between a visible and a hidden platform in the goal arm, with three hidden and three visible platform trials. Block 3 consisted of three trials with a hidden platform. For day 2, mice were tested in three blocks of five trials each (15 total trials), with only the hidden escape platform employed, forcing the mice to use a spatial strategy to identify the goal arm location. Data are presented as average errors per block. Only errors during the hidden platform trials represent the spatial memory component and are included in the analysis of the RAWM task.

### Statistical analysis

In the cross-sectional study design, experimental groups consisted of two genotypes, the Cntr and the *smpd3-/-* genotype at ages 3, 6, 12, 20, and 24 mo. Sizes of animal cohorts were six genders and weight matched mice, or otherwise listed under respective figures and Material and Methods. Results are expressed as mean ± SD. Statistical significance of differences between individual experimental groups was compared by the unpaired *t*-test using Graph Pad Quick Calcs: t-test calculator. *p* values of ≤ 0.05 *, ≤ 0.01 **, ≤ 0.001 *** were considered significant

## Electronic supplementary material

SMPD3 deficiency perturbs neuronal proteostasis and causes progressive cognitive impairment.
