# Supplementary material for: SMPD3 deficiency perturbs neuronal proteostasis and causes progressive cognitive impairment
Source: Cell Death Dis. 2018 May 3;9(5):507. doi: 10.1038/s41419-018-0560-7 (PMC5938706; doi:10.1038/s41419-018-0560-7)
Supplement: Supplementary file 1 — SMPD3 deficiency perturbs neuronal proteostasis and causes progressive cognitive impairment. [file 41419_2018_560_MOESM1_ESM.pdf]

**Supplementary Information**

# SMPD3 deficiency perturbs neuronal proteostasis and causes progressive cognitive impairment

Wilhelm Stoffel*[,1, 2, 3], Britta Jenke[2], Inga Schmidt-Soltau[2], Erika Binczek [1], Susanne Brodesser[3], Ina Hammels[1]

[1]Laboratory of Molecular Neuroscience, Institute of Biochemistry, University of Cologne, 50931 Cologne, Germany

[2]CMMC (Centre for Molecular Medicine), University of Cologne, 50931 Cologne, Germany

[3]CECAD (Cluster of Excellence: Cellular Stress Responses in Aging-Associated Diseases), University of Cologne, 50931 Cologne, Germany

## Method

## Mass Spectrometry

Dried lipid extracts were dissolved in 10 mM ammonium acetate in methanol and loaded into 96-well plates for the analysis of lipid subspecies by mass spectrometry. Lipid infusion and ionization was conducted using Nano-ESI chips with the TriVersa NanoMate operated by the ChipSoft Software (Advion) under the following settings: sample infusion volume: 14 µl, volume of air to aspirate after sample: 1 µl, air gap before chip: enabled, aspiration delay, 0s, pre-piercing, with mandrel, spray sensing, enabled, cooling temperature: 10°C, gas pressure, 0.5psi, ionization voltage: 1.4 kV, and vent headspace, enabled. Pre-wetting was done once. Mass spectrometric analysis was performed using the QTRAP 6500 (SCIEX) operated by Analyst 1.6.2. The following instrument-dependent settings were used: curtain gas, 20 psi; CAD gas, medium; and interface heater temperature, 100°C. PC and SM analysis was performed by scanning for precursor ion m/z 184 at a collision energy (CE) of 35 eV. PE, PS, and PI were analyzed by scanning for neutral losses of 141, 185, and 277 at a CE of 25 eV. Cer and GalCer species were detected by scanning for precursors of m/z 264 with a CE of 35 and 40 eV, respectively. The analysis of DAG species was conducted by scanning for the neutral losses of the ammonium adducts of distinct fatty acids with a CE of 25 eV: 273 (16:0),

297 (18:2), 299 (18:1), 301 (18:0), 321 (20:4), and 345 (22:6). All scans were conducted in the positive ion mode with a declustering potential of 100 V and at a scan rate 200 D/s (Ozbalci et al. 2013).

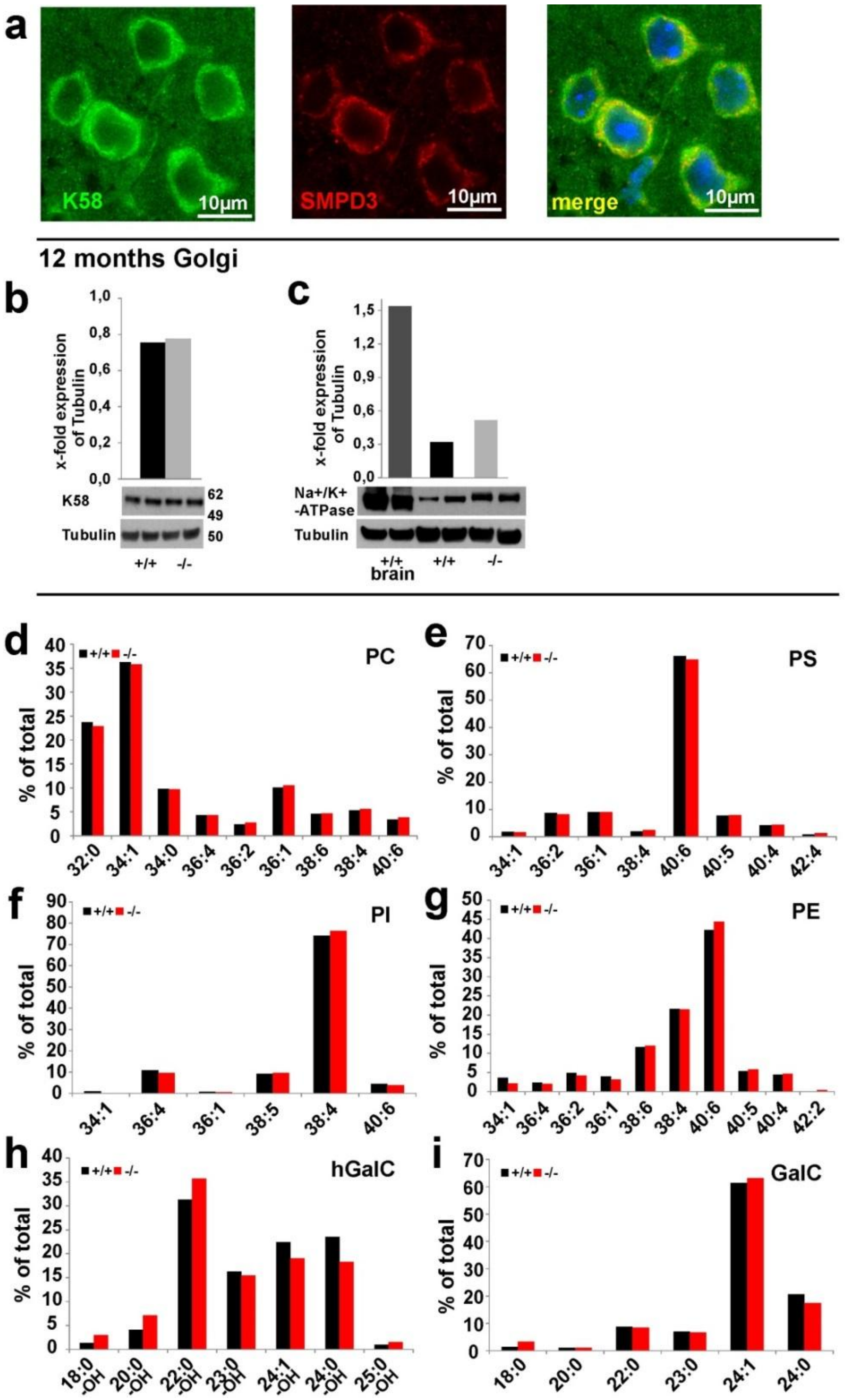


**Supplementary Figure 1** (**a**) Colocalization of SMPD3 and K58 in Golgi of cortex. Western blot analysis of the Golgi-fraction using (**b**) anti-K58 and (**c**) comparison of Cntr and *smpd3-/-* with lysate brain, using anti-Na+/K+-ATPase antibody. Lipidomic analysis of total lipid extract of Golgi membranes of Cntr and *smpd3-/-* brains. Species analysis of phospholipid classes by MS/MS of (**d**) phosphatidyl choline (PC), (**e**) phosphatidyl serine (PS), (**f**) phosphatidyl inositol (PI), (**g**) phosphatidyl ethanolamine (PE), and sphingolipids (**h**) 2-hydroxyacyl-galactocerebrosides (hGalC) and (**i**) galactocerebrosides (GalC).

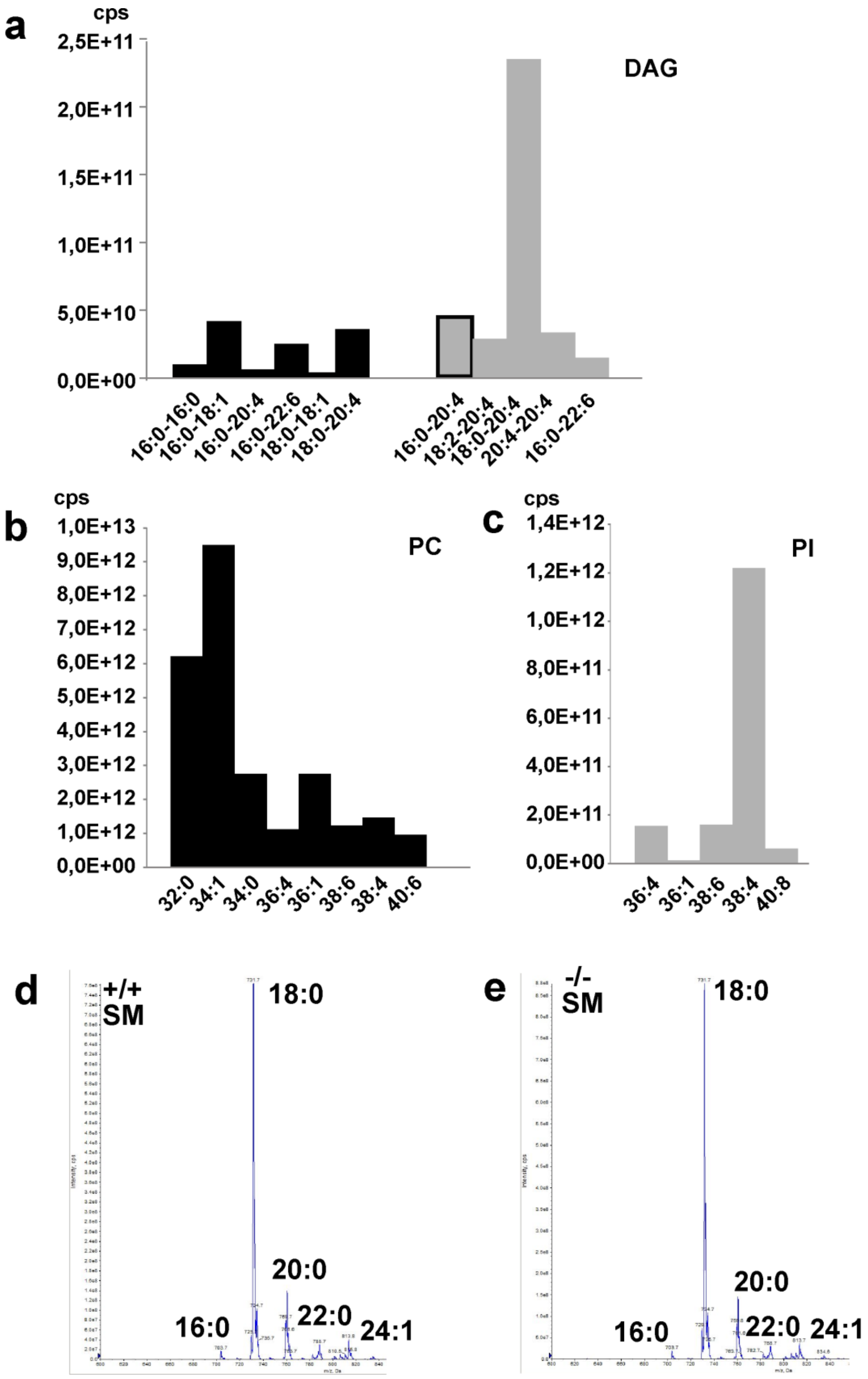


**Supplementary Figure 2** Comparison of DAG-species in DAG-pools. (**a**) Golgi-compartment with DAG-species of (**b**) PC- fraction and (**c**) PI-fraction, separated by HPTLC, identified and quantified by MS/MS. (**d, e**) The MS/MS specific spectra exemplify the SM fraction.

**TableS1**

| | | | |
|---|---|---|---|
| app s | 5'-agcttggcactgctcctgctg-3' | syntaxin s | 5'-gtttgtggaggtcatgtc-3' |
| app as | 5'-gtgtgtcttgcactgcttgcg-3' | syntaxin as | 5'-ctccagcttgatgatctc-3' |
| psen1 s | 5'-gagatacctgcacctttgtcc-3' | mbp s | 5'-cagagtccgacgagcttc-3' |
| psen1 as | 5'-cttccgggtatagaagctgac-3' | mbp as | 5'-gggtagttctcgtgtgtg-3' |
| psen2 s | 5'-ctcgcattcatggcctctgac-3' | ttbk 1 s | 5'-gcagtacactaacaccac-3' |
| psen2 as | 5'-cacagacttgatagtggccac-3' | ttbk 1 as | 5'-gtaatcgggcttggtgaa-3' |
| synaptophysin s | 5'-atgctgctgctggcagacatg-3' | plp 1 s | 5'-gttgtatggctcctggtg-3' |
| synaptophysin as | 5'-gcaggagggtgcatcaaagta-3' | plp 1 as | 5'-ggcgaagttgtaagtggc-3' |
| hgprt s | 5'-gctgacctgctggattacattaaagcactg-3' | glast1 s | 5'-gcaacggagaagagcctagga-3' |
| hgprt as | 5'-attcctgaagtactcattatagtcaagggc-3' | glast1 as | 5'-ggagggcaaatccaaggattg-3' |
| mapt s | 5'-gtggacctgagcaaagtgacc-3' | eaac s | 5'-cttcctacggaatcactggctgctgctctc-3' |
| mapt as | 5'-gagtccaccatgtcgatgctg-3' | eaac as | 5'-aacatggcatccatggtgctgacttcaggg-3' |
| synapsin s | 5'-gacggaagggatcacatt-3' | | |
| synapsin as | 5'-aagatgttgctggccttg-3' | | |

Ozbalci C, Sachsenheimer T, Brugger B. Quantitative analysis of cellular lipids by nano-electrospray ionization mass spectrometry. Methods Mol Biol 2013; 1033: 3-20.
